# The Formation Mechanism of a Self-Organized Periodic-Layered Structure at the Solid-(Cr, Fe)_2_B/Liquid-Al Interface

**DOI:** 10.3390/ma13173869

**Published:** 2020-09-02

**Authors:** Mengmeng Wang, Jiang Ju, Jingjing Li, Yang Zhou, Haiyang Lv, Haiyan Gao, Jun Wang

**Affiliations:** 1 School of Materials Science and Engineering, Shanghai Jiao Tong University, Shanghai 200240, China; mengmengwang@sjtu.edu.cn (M.W.); jujiang1990@sjtu.edu.cn (J.J.); lijingjing-41422134@sjtu.edu.cn (J.L.); yzhou76@sjtu.edu.cn (Y.Z.); lvhaiyang@sjtu.edu.cn (H.L.); 2 Shanghai Key Laboratory of Advanced High-Temperature Materials and Precision Forming, Shanghai Jiao Tong University, Shanghai 200240, China

**Keywords:** periodic-layered structure, self-assembly, interface diffusion, phase transformations, focused ion beam

## Abstract

A periodic-layered structure was observed in solid-(Cr, Fe)_2_B/liquid-Al diffusion couple at 750 °C. The interface morphology, the reaction products, and the potential formation mechanism of this periodic-layered structure were investigated using an electron probe microanalyzer (EPMA), scanning electron microscopy (SEM), electron backscatter diffraction (EBSD), transmission electron microscopy (TEM), and energy-dispersive spectroscopy (EDS). The results indicate that the reaction between (Cr, Fe)_2_B and liquid Al is a diffusion-controlled process. The formation of intermetallics involves both the superficial dissolution of Fe and Cr atoms and the inward diffusion of Al at the interface. The layered structure, as characterized by various experimental techniques, is alternated by a single FeAl_3_ layer and a (FeAl_3_ + Cr_3_AlB_4_) dual-phase layer. A potential mechanism describing the formation process of this periodic-layered structure was proposed based on the diffusion kinetics based on the experimental results.

## 1. Introduction

The periodic-layered structure (PLS) is a kind of in-situ nanocomposites, and has attracted much attention due to its interesting layered microstructure features and their potential application in novel energy conversion materials and electrocatalytic performance [1,2]. The PLS is a kind of self-organized structure formed by reaction-diffusion and has found in numerous alloy systems, such as Ni_3_Si/Zn [3,4,5,6], Cu_x_Ti_y_/Zn [7], SiO_2_/Mg [8,9,10], (Ni, W)/Al [11], U-Mo/Al [12], and so on. However, the discovery of PLSs are always occasionally, and the formation mechanism of PLS remains unclear, yet, sometimes, the existing formation mechanisms are contradictory to each other [13].

To explain the possible formation mechanisms of the PLS, numerous models have been developed. For the diffusion-induced stresses model describing the kinetic instability mechanism [6], the periodic layered structure was assumed as an alternation of a single-phase α layer and a (α + β) two-phase layer within the reaction zone. In some systems such as SiC/Ni [14] and Sn/(Ni-7%V) [15], however, the periodic layered structures were reported as a composition without any crack observed. The thermodynamic instability model assumed the PLS is the assembled layer containing a single α phase and a single β phase [6]. However, the thermodynamic instability model failed to explain the formation of periodic structure alternately by a single-phase layer and a two-phase layer. A diffusion-controlled precipitation and growth model developed by Su et al. [5] show that the mobility differences of the species at the interface have a key influence on the formation of the PLS. They further concluded that the slow diffusion species accumulate at the reaction front, and give rise to the precipitation of another phase within the matrix phase. It can be seen that the existing mechanisms always contradict each other regarding the situation that the PLS is alternated by a single-phase layer and a dual-phase layer.

The Fe-Cr-B cast steels have attracted increasing research interests due to their potential application in Al die-casting as anticorrosion and wear parts (e.g., the crucibles and pumps) [16]. Cast Fe-Cr-B steels consist of a microstructure of a ductile Fe-based matrix and large fractions of borides. The borides, typically have formula as (Fe, Cr)_2_B or (Cr, Fe)_2_B, possess high hardness [17], a modulus [17], and good corrosion resistance to the Al melts [18]. Recently, Zhang et al. [13] observed the formation of PLS in the solid-(Cr, Fe)_2_B/liquid-Al couple at 750 °C. They claimed that the PLS formed at the solid-(Cr, Fe)_2_B/liquid-Al consists of alternating layers of the Fe-Al phase and a kind of ternary Cr-Al-B phase. However, more reliable identification of the constituent phases within the (Cr, Fe)_2_B/liquid-Al couple was not available at the time, and the formation mechanism of the PLS remains unclear.

The clarification of the formation mechanism of PLS allows the creation of novel periodic layered structures and explores novel Fe-Cr-B alloys for industrial application. In this study, we therefore systematically investigate the interface morphology, reaction products within the reaction layer, as well as on the potential formation mechanism of the PLS at the solid-(Cr, Fe)_2_B/liquid-Al interface, based on the experimental findings.

## 2. Material and Methods

### 2.1. Preparation of Samples

The bulk Fe-Cr-B alloy was prepared by repeated argon arc melting from purity chromium (99.99 wt.%), pure iron (99.99 wt.%), and commercial Fe-17 wt.% B master alloy. The mixtures of elements of formula proportion were melted at least four times to ensure the homogeneity of the compositions in a water-cooled copper crucible with argon gas atmosphere. The composition of the Fe-Cr-B alloy (28.92% Fe, 35.85% Cr, 33.19% B, and 2.04% Si in at.%) was measured by inductively coupled plasma-atomic emission spectroscopy (ICP-AES, ICP 6300, Thermo-Scientific, Waltham, MA, USA). The sample with a dimension of 15 × 9 × 4 mm^3^ for the immersion test was cut from the center of the cast billet using the electric discharge machine (EDM).

### 2.2. Immersion Test

The immersion test against liquid Al (99.99 wt.% pure) was performed in a graphite crucible placed in an electric resistance furnace. The bath temperature was set to 750 °C and the temperature was monitored by an external thermocouple protected by an alumina tube during the immersion test. The Fe-Cr-B alloy was immersed in liquid Al at 750 °C for 8 h, and the apparatus for the immersion test was illustrated in detail in our previous study [19,20]. After the immersion test, the specimen was withdrawn from the Al bath and cut perpendicular to the solid/liquid interface for metallographic observation using the standard grinding and polishing.

### 2.3. Material Characterization

Microstructure of the as-cast Fe-Cr-B alloy was characterized by a JSM 7600F (JEOL, Tokyo, Japan) scanning electron microscope (SEM) equipped with an energy-dispersive X-ray spectroscopy (EDS; Oxford X-MaxN80, Oxford-instruments, UK) and a JXA-8230 electron probe microanalyzer (EPMA; JEOL, Tokyo, Japan) with wavelength-dispersive spectroscopy (WDS). The standard reference materials used for EPMA measurement were pure elements, and the adopted beam condition was 10–15 kV, and 60–100 nA. For the EPMA analysis, at least five concentration profiles were averaged to reduce statistic errors.

The PLS presents a nanosized microstructure [13], which is unable to be analyzed by EPMA with 1 μm-resolution. Therefore, the chemical compositions of the phases in the PLS were analyzed using TEM-EDS (SuperX G2; FEI, Hillsboro, OR, USA) under the STEM-HAADF (scanning transmission electron microscopy-high angle annular dark field) mode performed on a FEI Titan ETEM G2 (FEI, Hillsboro, OR, USA) spherical aberration-corrected transmission electron microscope (TEM). Thin foil specimen for TEM observation was prepared using a focus ion beam (FIB) lift-out technique. The observed area of interest contains two layers of (Cr, Fe)_2_B matrix and the PLS. A Pt layer was deposited on the specific area to protect the surface and the milled face from damage induced by ion. A deep trough was drilled using the Pt-deposited layer as a reference. The specimen was finally mounted on the Cu half grid and ion-milled to acquire a thin film for TEM observation.

Crystal structures of the phases were further determined using electron backscattered diffraction (EBSD) technique. The EBSD sample was prepared by a technique of ion beam slope cutting with a Leica EMTIC 3X instrument (Leica, Germany). The EBSD analyses were performed on a Mira3 SEM with a high speed EBSD detector (Oxford Nordly Max3, oxford-instruments, UK)), and the step size for EBSD measuring was set to 10 nm, the lower limit of this apparatus. In present work, more than 10 diffraction bands were required for phase identification.

## 3. Results and Discussion

### 3.1. As-Cast Microstructure

**Figure 1:**
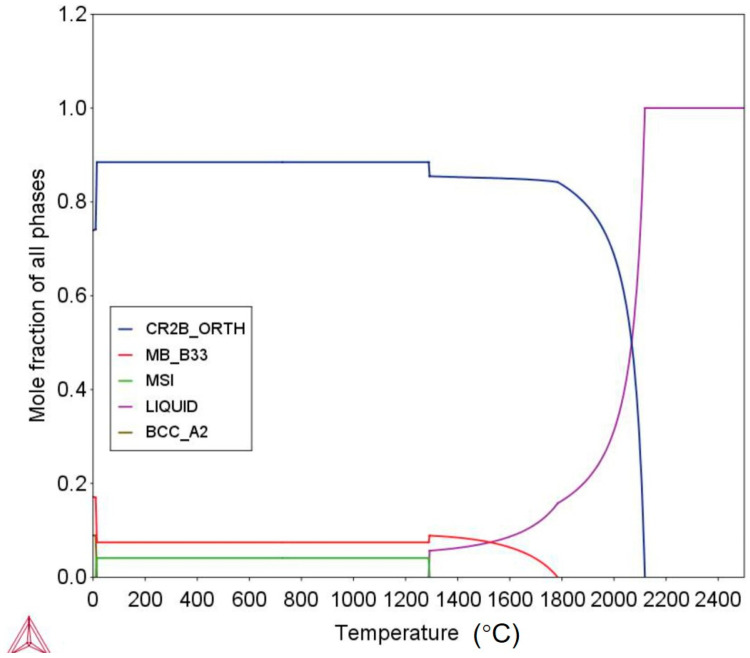
THERMOCALC calculations of the phase equilibrium for the alloy used in the current study.

**Figure 2:**
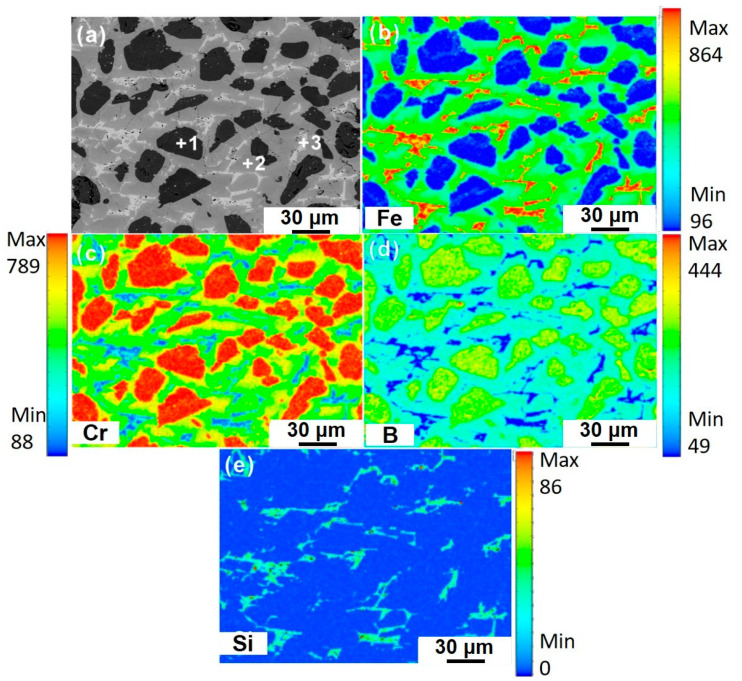
(**a**) The as-cast microstructure of the Fe-Cr-B alloy. (**b**–**e**) The electron probe microanalyzer (EPMA) elemental mappings of the as-cast microstructure.

THERMOCALC^®^ software (Version2017b) developed by the CALPHAD technique was used to calculate the potential phases that could form in the as-cast Fe-Cr-B alloy. Figure 1 shows the phase equilibrium of the Fe-Cr-B alloy using the TCFE8 database designed for iron-based alloys. As can be seen from Figure 1, the orthorhombic Cr2B, MB, and MSi essentially dominate the microstructure, while the BCC-A2 phase is more stable at a lower temperature. The BCC-A2 phase is a α-Fe solid solution, where Cr and Si are the substitutional elements, and B is in the form of interstitial atom. Figure 2 shows the SEM micrograph and the EPMA elemental mappings of the as-cast microstructure of the Fe-Cr-B alloy. It can be seen that the as-cast microstructure of the Fe-Cr-B alloy mainly consists of three phases with distinguishable compositional contrast, specifically, the light-colored matrix, the black globular phase, and the white grainy phase. It is clear from the maps, that the globular phase is enriched in B and Cr, while there is less partitioning of Fe. The Cr, Fe, and B elements have considerable contents in the light-colored matrix, while the grainy phase is enriched in Fe and Si with B depleted. The EPMA quantitative analysis was performed on the individual phases (numbered as 1–3 in Figure 2a), and the mean composition of individual phases were listed in Table 1. From point 1, it is clear that the Cr and B ratio of the globular phase is very close to 1. The result from point 2 indicating that the matrix has a formula Fe_0.99_Cr_1.18_B, which may be much closer to the structure of M_2_B (where M represents Fe and Cr) boride, typically as the CrFeB boride [21]. The composition results from point 3 indicating the grainy phase is much close to the Fe-based solid solution by comparing with the reference [22], where the Cr, Si, and B are the dissolved elements. Combined with the thermodynamic calculations, it becomes evident that the light-colored matrix, the black globular phase, and the grainy white phase should correspond to the orthorhombic (Cr, Fe)_2_B boride, CrB boride, and the α-(Fe, Cr, Si) solid solution.

**Figure 3:**
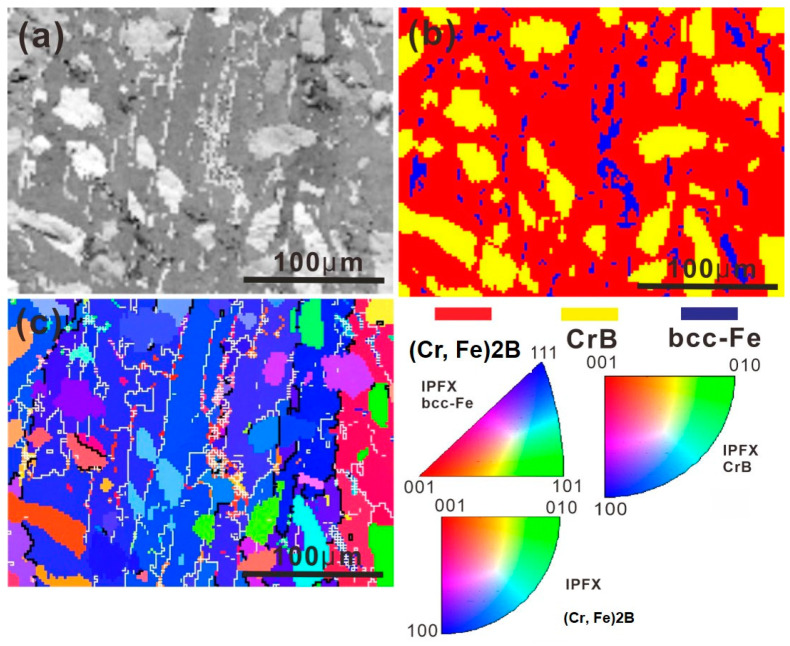
Electron backscattered diffraction (EBSD) analysis of the as-cast microstructure in the Fe-Cr-B alloy: (**a**) the band contrast map; (**b**) phase distribution map; and (**c**) the inverse pole figure (IPF) map.

The constituent phases in the as-cast microstructure were further analyzed by EBSD based on their crystal structure (the crystal structure of individual phases used in the EBSD measurement was listed in Table 2). The characterization results from the EBSD of the as-cast microstructure are shown in Figure 3a–c. Figure 3a is the band contrast map of the as-cast microstructure. It is shown in Figure 3b, the light-colored matrix, black globular phase, and the white grainy phase correspond to (Cr, Fe)_2_B, CrB, and α-Fe phase, respectively. The (Cr, Fe)_2_B phase has an orthorhombic structure as the Cr_2_B phase, and can be regarded as a result of the replacement of Cr by Fe atoms in the lattice of Cr_2_B [17]. The inverse pole figure (IPF) map of the as-cast microstructure is shown in Figure 3c, and the thick black lines and thin white lines correspond to the high angle grain boundaries (HAGBs, misorientation angle >15°) and the low angle grain boundaries (LAGBs, misorientation angle within 2–15°), respectively. The equivalent mean grain diameter of (Cr, Fe)_2_B, CrB, and α-Fe was calculated by OIM software to be 52.44 ± 26.78 μm, 24.19 ± 8.5 μm, and 5.33 ± 1.98 μm, respectively. It can be seen that the (Cr, Fe)_2_B phase has rather a coarse grain size. Considering the nanoscaled nature of the PLS [13], the interface reaction can be considered as the reaction between a bulk crystal and liquid Al.

### 3.2. Interface Morphology and Reaction Products

**Figure 4:**
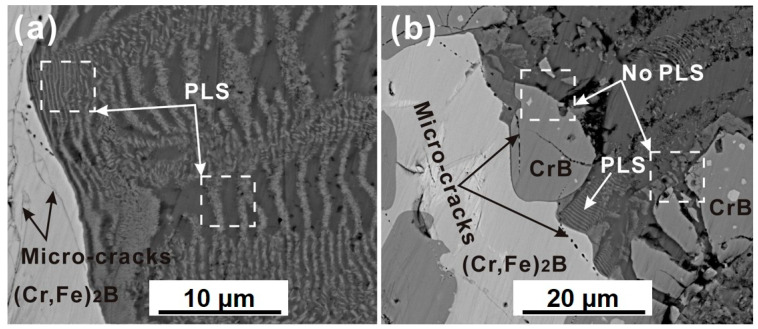
The cross-sectional morphology of the Fe-Cr-B alloy in liquid Al at 750 °C for 8 h. (**a**) The enlarged view of interface morphology at the (Cr, Fe)_2_B/Al interface. (**b**) The interface morphology observed at the CrB/Al interface.

The cross-sectional micrographs of the specimen after immersion in liquid Al for 8 h were shown in Figure 4. As seen in Figure 4a, the PLS was observed at the solid-(Cr, Fe)_2_B/liquid-Al interface, and the layered structure is nearly parallel to the interface. Microcracks were observed at the (Cr, Fe)_2_B phase near the interface, as shown in Figure 4b. It is generally accepted that the initiation of the interface crack may be caused by either the influence of residual stress or thermal change in the coating/matrix system. Energy can be released by generating the cracks when the thermal changing due to the difference of the thermal coefficient between matrix and coating. Moreover, from the selected zones in Figure 4, it is clear that the PLS has undergone a splitting process and finally forms new layers with much smaller layer spacing. This phenomenon will be discussed in detail in the following sections (see Section 3.4).

**Figure 5:**
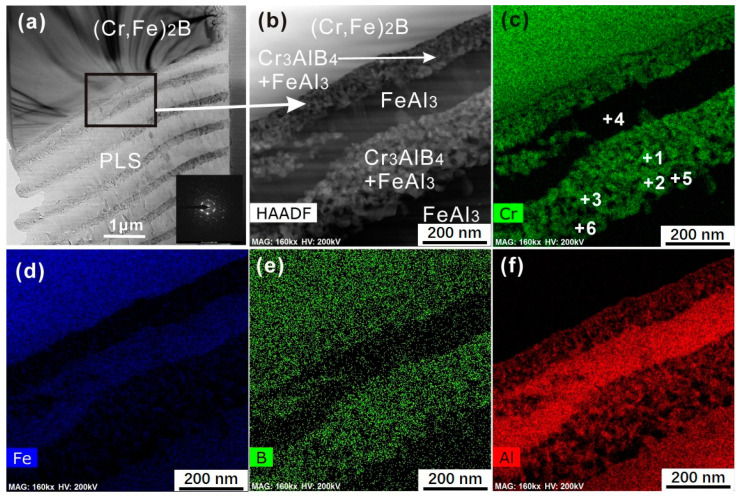
(**a**) The TEM micrograph of the periodic-layered structure (PLS). (**b**) The scanning transmission electron microscopy (STEM)-HADDAF micrograph of the PLS. (**c**–**f**) The elemental distribution of individual phases in the PLS.

**Figure 6:**
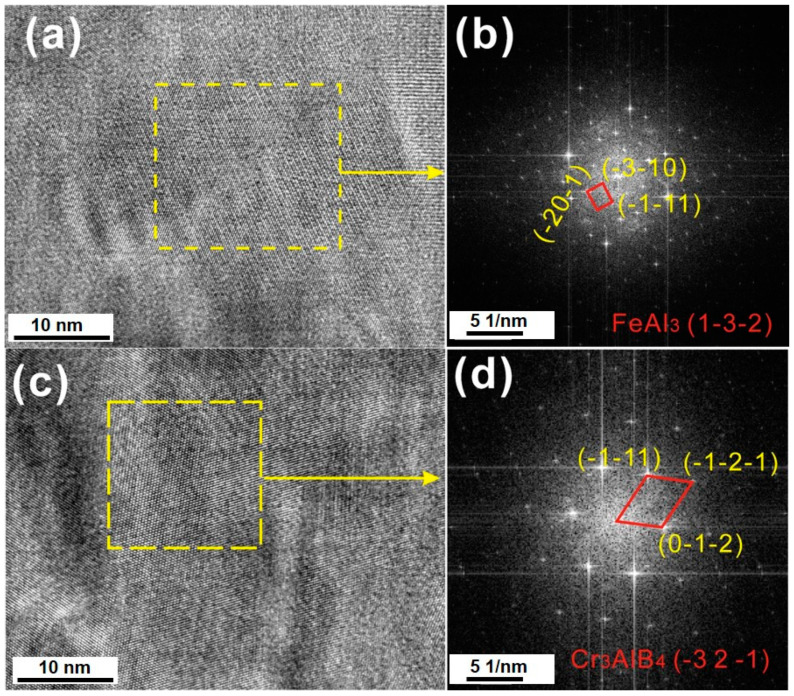
(**a**,**c**) The high resolution TEM (HRTEM) micrographs taken from the Cr-rich zone and the Cr-depleted zone within the Cr-rich band. (**b**,**d**) The fast Fourier transform (FFT) graphs correspond to (**a**,**c**), respectively.

Thin foil was prepared by the FIB lift-out technique for the TEM observation, which includes two distinct layers of (Cr, Fe)_2_B and PLS. Figure 5 shows the TEM micrograph and the elemental mappings of the individual elements in the (Cr, Fe)_2_B/Al diffusion couple. The STEM-HAADF micrograph of PLS shown in Figure 5b indicates that the thin layer composited fine grains with a grain size around tens of nanometers, while the thick layer represents column morphology and a larger grain size. Figure 5c–f shows the STEM-HADDF mappings of the PLS, and it is clear that the thin layer was Cr-rich and the thick layer was Cr-depleted and Al-enriched. Interestingly, it seems that the distribution of individual elements in the Cr-rich layer were not homogenous. More specifically, in this layer, the Cr-depleted zones were enriched in Fe and Al, while the Cr-rich zones containing high B contents with Al and Fe depleted. The TEM-EDS analysis was performed on the interface at these locations numbered as 1–6 in Figure 5c. The Cr-rich zones are marked as 1–3, while that of the Cr-depleted zones are marked as 4–6. The results from EDS were shown in Table 3. The crystal structure of Cr-rich and Cr-depleted phases was further determined by HR-TEM (high resolution-TEM) micrographs and the diffraction patterns from the corresponding fast Fourier transform (FFT; as seen in Figure 6). From the FFT graphs (Figure 6b,d), the FeAl_3_ phase and ternary Cr_3_AlB_4_ phase were indexed.

However, the phase constituents of the PLS cannot be confirmed only from the TEM results, because the TEM observation was from the microscopic view. According to the Al-rich corner of the Fe-Al binary diagram [31], the FeAl_3_ phase and Fe_2_Al_5_ phase coexisted at 750 °C, that is, the Fe-Al phase in the PLS might be one of them or perhaps two of them from the thermodynamic view. Besides, according to the isothermal section of the ternary Cr-Al-B diagram at 700 °C [32], there were three kinds of Cr-Al-B intermetallics, specifically, Cr_3_AlB_4_, Cr_2_AlB_2_, and Cr_4_AlB_6_ phases. Considering the measurement error caused by the TEM-EDS method and the similar atomic ratios, reaching a phase by TEM-EDS seems unreasonable.

**Figure 7:**
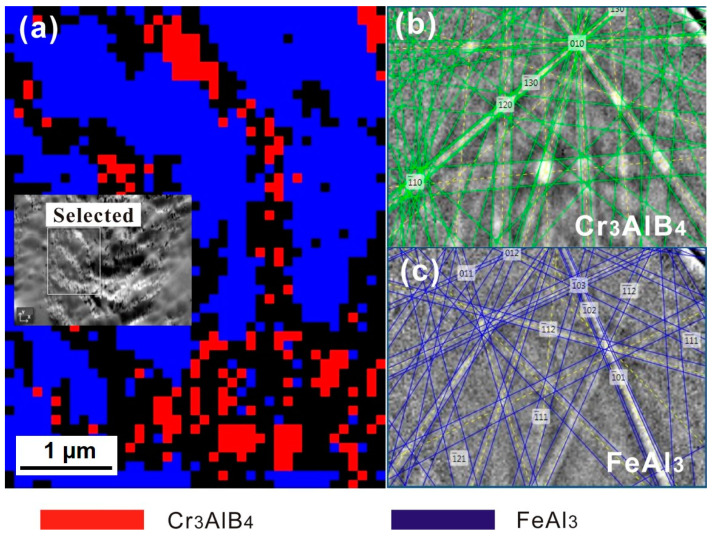
EBSD mappings of the phase distribution in the corrosion layer: (**a**) phase distribution map. (**b**,**c**) Kikuchi patterns from individual phases.

EBSD analysis was utilized to distinguish the constituent phases within the PLS from a more macroscopic view. The crystal structures of the potential phases in the PLS was given in Table 2. The crystal information files were input to the EBSD software before the EBSD measurement. Figure 7 is the phase distribution map from the EBSD analysis. The inserted micrograph in Figure 7a shows the SEM image of the PLS. In Figure 7a, the blue and red correspond to the FeAl_3_ phase and the Cr_3_AlB_4_ phase, respectively. Figure 7b,c corresponds to the Kikuchi patterns obtained from the FeAl_3_ phase and Cr_3_AlB_4_ phase. It is clear, no other ternary Cr-Al-B phase was detected in the PLS, referring that the Cr-Al-B phase in the PLS belongs to the Cr_3_AlB_4_ phase. Moreover, the Fe_2_Al_5_ phase was not detected in the PLS, indicating that the Fe-Al phase belongs to the FeAl_3_ phase.

The results from both TEM and EBSD indicates that FeAl_3_ grains and Cr_3_AlB_4_ grains coexisted in the dual-phase layer, which proves that the PLS is not alternated by two single phases, but composed of a single phase FeAl_3_ layer and a dual-phase (Cr_3_AlB_4_ + FeAl_3_) layer.

### 3.3. Diffusion Kinetics of an Individual Element

**Figure 8:**
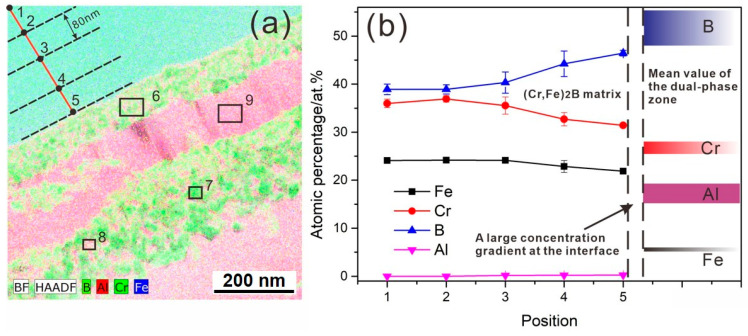
(**a**) STEM-HAADF mappings of the interface. (**b**) The measured concentration profiles along the red line.

As shown in Figure 8, a concentration profile along the red segment was measured using TEM-EDS with a step size of 80 nm for evaluation of the concentration variation of individual elements (e.g., Fe, Cr, B, and Al) in the (Cr, Fe)_2_B phase. At least three points were performed at each position in order to obtain the average concentration values at some individual distance from the interface (as the black dash lines, which parallel to the interface). The corresponding analysis data was shown in Figure 8b and Table 4. For estimating the accuracy of measurement data, the standard deviation is given in the table. From Figure 8b, it is clear that the diffusion depth was approximately 320 nm, which is much less than the 1 μm-lateral resolution of the EPMA, so EPMA analysis was not available here. Moreover, it can be seen that Fe and Cr elements shows a pronounced decrease within the diffusion layer, indicating that the reaction is a strong dissolution process of Fe and Cr elements. B element shows an increase from the matrix side to the interface. Al diffuses toward the (Cr, Fe)_2_B matrix with concentration decreasing from the Al-rich side to the matrix side.

The results indicate that, at the initial stage, the reaction between (Cr, Fe)_2_B and liquid Al is a dissolution process involving Fe and Cr, accompanied by a strong Al inward diffusion. At the same time, the FeAl_3_ phase and the Cr_3_AlB_4_ phase precipitate at the interface, thus a thin film of dual-phase zone forms. As times goes on, the dual-phase zone grows thicker, and the (Cr, Fe)_2_B/(FeAl_3_ + Cr_3_AlB_4_) interface push towards the (Cr, Fe)_2_B matrix side owing to the Kirkendall effect [10]. The average composition of the dual-phase zone was analyzed, as shown in Table 4 (point 6). The colored bar in Figure 8b represent the average composition of the dual phase zone, it is clear that a large composition gradient of Fe and Al existed at the interface, thus promoting the nucleation of FeAl_3_ phase. Meanwhile, as the two-phase layer of (Cr_3_AlB_4_ + FeAl_3_) phase grows thicker and thicker, Fe and Al interdiffusion will be retarded by the thicker two-phase layer, which would promote the nucleation of the FeAl_3_ phase.

### 3.4. The Potential Formation Mechanism

**Figure 9:**
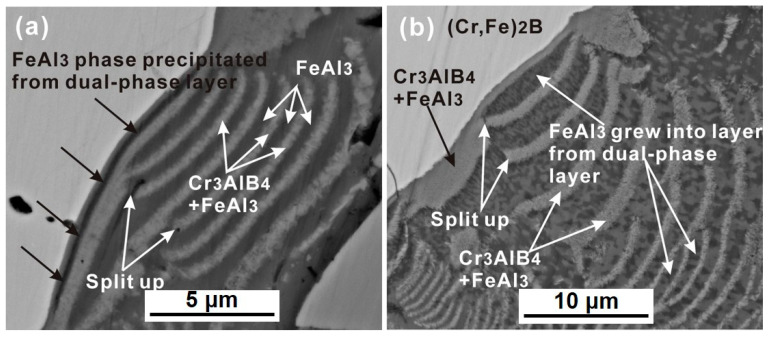
The high-resolution SEM micrographs of the reaction interface within the reaction zone. (**a**) FeAl_3_ phase precipitated from the dual-phase layer. (**b**) FeAl_3_ grew into new layer from dual-phase layer.

Figure 9 shows the enlarged cross-sectional micrographs of the (Cr, Fe)_2_B/Al couple at 750 °C. From the arrowed region shown in Figure 9a, a thin FeAl_3_ layer was precipitated from the (Cr_3_AlB_4_ + FeAl_3_) dual-phase layer. As time goes on, the FeAl_3_ layer grew thicker and developed into layer-like morphology within the dual-phase layer (as seen in Figure 9b).

As mentioned in the introduction section, there might be two different mechanisms for PLS alternated by a single-phase layer and a dual-phase layer, namely, the diffusion-induced stresses model and the diffusion-controlled precipitation and growth model. In the diffusion-induced stresses model [6], it was assumed that the single-phase layer and dual-phase layer form at the same time at the reaction front in the initial stage, which is not in agreement with the present experiment results, where the single-phase FeAl_3_ layer is formed by conjunction precipitated FeAl_3_ phase in the dual-phase layer (as seen in the arrowed region in Figure 9).

**Figure 10:**
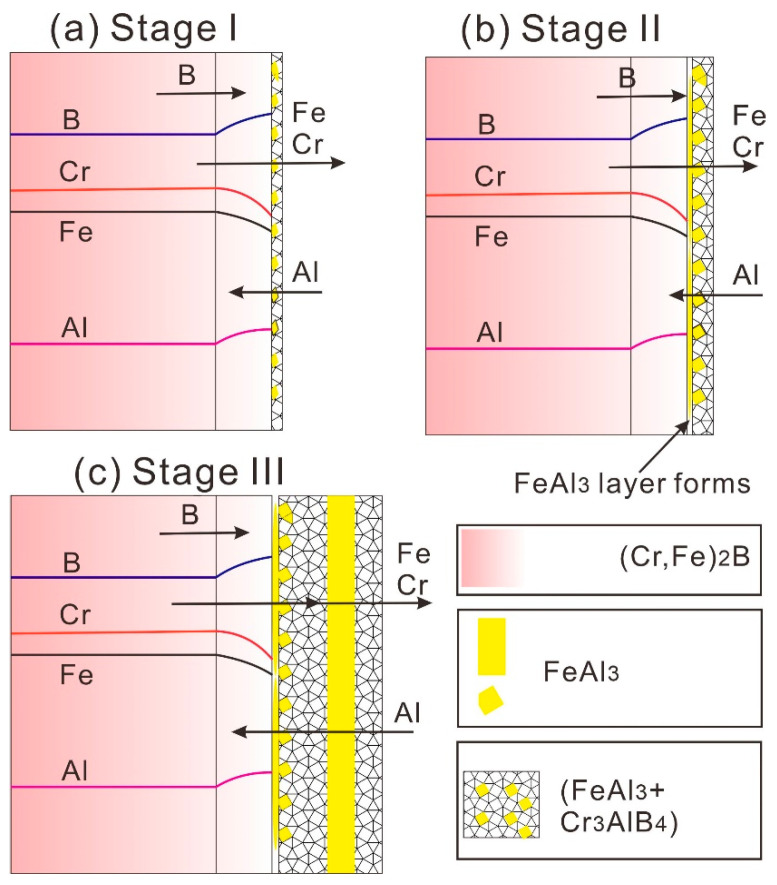
The schematic drawings showing the formation of the periodic-layered structure by the solid-(Cr, Fe)_2_B/liquid-Al reaction. (**a**) A thin (Cr_3_AlB_4_ + FeAl_3_) dual-phase layer was formed. (**b**) FeAl_3_ grains conjunct into the thin plate within the dual-phase layer. (**c**) A new periodic pair is formed.

Based on the present experimental findings, the formation mechanism of the periodic-layered structure at the (Cr, Fe)_2_B/Al interface supports the diffusion-controlled precipitation and growth model, and the mechanism is graphically illustrated in Figure 10. The growth in the PLS at the (Cr, Fe)_2_B/Al interface involves inward diffusion of Al, dissolution of Fe and Cr. The growth of the FeAl_3_ phase was supported by the diffusion of Al from the Al-rich side to the (Cr, Fe)_2_B/(Cr_3_AlB_4_ + FeAl_3_) interface, and the growth of Cr_3_AlB_4_ phase was sustained by the diffusion of Fe and Cr from the (Cr, Fe)_2_B matrix, the diffusion of Al from the Al-rich side, as well as the fast diffusion of B (Figure 10a). With immersion time going on, the two-phase layer of (Cr_3_AlB_4_ + FeAl_3_) phase grows thicker and thicker, Fe and Al interdiffusion will be retarded by the thicker two-phase layer. Meanwhile, a great Al gradient was built at the (Cr, Fe)_2_B/(FeAl_3_ + Cr_3_AlB_4_) interface, thus the FeAl_3_ phase is in favor to nucleate and grows within the short-range of Fe diffusion (Figure 10b). After that, the small FeAl_3_ grains merged into large grains and conjunct into the thin plate to alleviate the interfacial energy, as illustrated in Figure 10b. When the FeAl_3_ phase conjunct together to form a new layer, a new periodic pair will form (Figure 10c).

## 4. Conclusions

In present work, a self-organized periodic layered structure (PLS) was observed in the solid-(Cr, Fe)_2_B/liquid-Al couple at 750 °C, and the formation mechanism as well as the constituent phases within the PLS were illuminated. The obtained conclusions are summarized as follows.

(i) A novel Fe-Cr-B alloy was prepared, consisting of (Cr, Fe)_2_B boride, the globular CrB phase, and the grainy α-Fe phase.
(ii) The PLS structure was observed at the Cr, Fe)_2_B/Al interface at 750 °C for 8 h, and was not found in that of CrB and α-Fe phases. The PLS within the reaction zone of the solid-(Cr, Fe)_2_B/liquid-Al couple was alternated by a single FeAl_3_ layer and a (Cr_3_AlB_4_ + FeAl_3_) dual-phases layer.
(iii) Based on the present experimental results, the periodic layer formation in the (Cr, Fe)_2_B/Al couple supports the diffusion-controlled precipitation and growth model. The formation of PLS undergoes three stages. At first, the preferential leaching of Fe and Cr elements from the (Cr, Fe)_2_B phase, accompanied by the inward diffusion of Al and fast diffusion of B toward the interface, resulting in the initial formation of the (Cr_3_AlB_4_ + FeAl_3_) dual-phase layer. As time goes on, the FeAl_3_ crystals precipitated from the Cr_3_AlB_4_ phase and growth into the new FeAl_3_ layer. Finally, the Cr-rich layer was split up to form new pairs.

## Figures and Tables

**Table 1:** The EPMA results of the selected region marked in Figure 2 (in at.%, standard deviation given in parentheses).

Points	Fe	Cr	B	Si
1	5.23(0.41)	46.82(0.34)	47.95(0.37)	-
2	31.11(2.11)	37.35(2.39)	31.54(0.29)	-
3	78.55(1.12)	9.81(1.49)	3.35(0.22)	8.29(0.85)

**Table 2:** The crystal structure of phases used for PLS identification by EBSD.

Phase	Cell Parameters					Structure		
	a(Å)	b(Å)	c(Å)	α	β	γ	Structure	Space Group
CrFeB [23]	14.57	7.32	4.22	90°	90°	90°	Orthorhombic	70
CrB [24]	2.98	7.87	2.93	90°	90°	90°	Orthorhombic	63
Fe(bcc) [25]	2.87	2.87	2.87	90°	90°	90°	Cubic	229
FeAl_3_ [26]	15.49	8.08	12.47	90°	90°	90°	Monoclinic	12
Fe_2_Al_5_ [27]	7.66	6.42	4.22	90°	90°	90°	Orthorhombic	63
Al [28]	4.05	4.05	4.05	90°	90°	90°	Cubic	225
Cr_3_AlB_4_ [29]	2.96	2.98	8.05	90°	90°	90°	Orthorhombic	47
Cr_4_AlB_6_ [29]	2.95	21.28	3.01	90°	90°	90°	Orthorhombic	65
Cr_2_AlB_2_ [30]	2.94	2.94	2.94	90°	90°	90°	Orthorhombic	65

**Table 3:** The TEM-EDS results of the selected regions marked in Figure 5 (in at.%).

Points	Fe	Cr	B	Al
1	6.15(0.35)	24.89(1.16)	52.50(3.28)	16.46(1.68)
2	4.18(0.41)	29.49(1.35)	51.45(3.09)	14.88(2.88)
3	5.08(0.38)	22.65(1.45)	55.13(2.86)	17.14(1.98)
4	22.63(2.18)	3.41(0.28)	-	73.96(2.45)
5	22.51(2.25)	5.42(0.22)	1.55(0.22)	70.52(3.2)
6	24.39(2.12)	4.14(0.19)	1.37(0.25)	70.10(2.66)

**Table 4:** Mean values of measured concentration profiles at locations marked in Figure 8 by TEM-EDS (in at.%, standard deviation given in parentheses).

Points	Fe	Cr	B	Al
1	24.10(0.33)	35.98(0.89)	38.92(1.08)	0
2	24.16(0.28)	36.93(0.67)	38.91(0.97)	0
3	24.13(0.42)	35.54(1.79)	40.33(2.20)	0.17(0.05)
4	22.85(1.25)	32.7(1.39)	44.24(2.65)	0.21(0.02)
5	21.89(0.42)	31.41(0.15)	46.47(0.58)	0.23(0.03)
6	5.17(0.42)	26.51(1.28)	51.44(3.72)	16.88(2.07)
7	2.31(0.60)	29.52(0.51)	61.04(1.08)	7.13(0.87)
8	20.24(3.89)	3.17(0.23)	-	76.59(3.70)
9	23.31(2.30)	3.87(0.23)	-	72.82(2.58)
